# Identification and prediction of alternative transcription start sites that generate rod photoreceptor-specific transcripts from ubiquitously expressed genes

**DOI:** 10.1371/journal.pone.0179230

**Published:** 2017-06-22

**Authors:** Evgenya Y. Popova, Anna C. Salzberg, Chen Yang, Samuel Shao-Min Zhang, Colin J. Barnstable

**Affiliations:** 1 Department of Neural and Behavioral Sciences, Penn State University, College of Medicine, Hershey, Pennsylvania, United States of America; 2 Penn State Hershey Eye Center, Hershey, Pennsylvania, United States of America; 3 Bioinformatics Core, Penn State University College of Medicine, Hershey, Pennsylvania, United States of America; University of Florida, UNITED STATES

## Abstract

Transcriptome complexity is substantially increased by the use of multiple transcription start sites for a given gene. By utilizing a rod photoreceptor-specific chromatin signature, and the RefSeq database of established transcription start sites, we have identified essentially all known rod photoreceptor genes as well as a group of novel genes that have a high probability of being expressed in rod photoreceptors. Approximately half of these novel rod genes are transcribed into multiple mRNA and/or protein isoforms through alternative transcriptional start sites (ATSS), only one of which has a rod-specific epigenetic signature and gives rise to a rod transcript. This suggests that, during retina development, some genes use ATSS to regulate cell type and temporal specificity, effectively generating a rod transcript from otherwise ubiquitously expressed genes. Biological confirmation of the relationship between epigenetic signatures and gene expression, as well as comparison of our genome-wide chromatin signature maps with available data sets for retina, namely a ChIP-on-Chip study of Polymerase-II (Pol-II) binding sites, ChIP-Seq studies for NRL- and CRX- binding sites and DHS (University of Washington data, available on UCSC mouse Genome Browser as a part of ENCODE project) fully support our hypothesis and together accurately identify and predict an array of new rod transcripts. The same approach was used to identify a number of TSS that are not currently in RefSeq. Biological conformation of the use of some of these TSS suggests that this method will be valuable for exploring the range of transcriptional complexity in many tissues. Comparison of mouse and human genome-wide data indicates that most of these alternate TSS appear to be present in both species, indicating that our approach can be useful for identification of regulatory regions that might play a role in human retinal disease.

## Introduction

The most basic identifier of a cell or tissue type is given by its transcriptome. However, the actual diversity of the proteome of a cell is much greater than the 20,000 to 30,000 protein-coding genes found in the mammalian genome. This diversity is achieved by a variety of mechanisms including alternative initiation and termination of transcripts, as well as posttranscriptional processes such as alternative splicing. Although alternative splicing plays a key role in many tissues, particularly the nervous system, recent studies have demonstrated that alternative initiation and termination have a greater contribution to transcriptome diversity [1–3]. Approximately half of mouse and human genes have alternative TSS and promoters [4–6]. Though some of the alternative TSS generate novel proteins, many result in identical open reading frames but different 5’-untranslated regions. Since the 5’-regions of mRNA contain binding sites for regulatory proteins, the use of alternative TSS can provide different pathways for controlling mRNA stability and translation [7–9]. More importantly, the different TSS have different promoters and thus the potential for different sets of transcription factors to regulate the temporal and spatial patterns of transcription. As well as different core promoter structures, alternative TSS can involve different chromatin/epigenetic state and histone modifications. Recent genome-wide studies that mapped different chromatin/ epigenetic features found that alternative TSS/promoters have distinctive chromatin states [1, 10, 11]. Conversely, chromatin/ epigenetic signatures can be used for prediction of new TSS and their usage in specific cell type or tissue [5, 12–14].

We have been using the mouse retina as a model system to understand the regulation of gene expression during development of different cell types from common progenitors. Our current understanding of the retina transcriptome has been derived initially from studies based on expression arrays [15–23] that lack information about transcription from alternative TSS, and more recently from RNA-seq data that still counts expression per gene, not for separate gene transcripts [24], even though the RNA-seq method could provide expression per isoform as well as per gene. Methods based on cap analysis of gene expression (CAGE) utilize trapping of RNA cap on 5’ end of transcript have been used to locate TSS in the genome [25–27]. Accurate positional information on TSS has been collected genome-wide for several tissues and cell types and is available in the Database of TSS (DBTSS) [26, 28, 29]. One CAGE based study for retina, was recently published, but focused on axonal injury and lacked information on the developmental aspects of retina [30].

In an alternative approach, chromatin features and epigenetic signatures can be used as predictors for gene expression status and expression levels [14]. Recently we used ChIP-seq to map the distribution of two important histone H3 modifications, H3K4me2 and H3K27me3, over the whole genome at multiple time points during late mouse retina development [31]. We merged these data with our previous retina developmental expression profiles [16] to define a striking epigenetic signature in genes known to generate a rod photoreceptor-specific transcript (termed “known rod genes” in this manuscript). We then used this signature in an unbiased search of the whole genome and identified a total of 107 genes: 36 known rod genes as well as a group of 71 novel genes (termed “new rod genes” in this manuscript) that we hypothesized have a high probability of generating transcripts in rod photoreceptors that are not found in other cell types.

In the present manuscript we have shown that many of these novel genes are transcribed into multiple mRNA or protein isoforms, using one TSS to generate a transcript in rod photoreceptors and another to generate a transcript in a broader array of cell types. We have also compared our genome-wide chromatin signature maps with ChIP-Seq studies for binding sites for CRX and NRL, two of the best characterized transcription factors for rod photoreceptor genes [32, 33], as well as available data sets for retina ChIP-on-Chip studies of Polymerase-II (Pol-II) binding sites [34] and DNase I hypersensitive sites (DHS), specific sites in genome where chromatin is more open and accessible (University of Washington data, available on UCSC mouse Genome Browser as a part of ENCODE project)[35]. This comparison fully supports our hypothesis that cell type restricted TSS can be determined and predicted by specific epigenetic signatures.

## Materials and methods

### Reagents

All chemicals were purchased from Fisher Scientific (Pittsburgh, PA), unless otherwise noted.

### Animals

All animal experiments were conducted in accordance with NIH guidelines and were approved by the Animal Care and Use Committee of Pennsylvania State University School of Medicine (Protocol # 46432). C57BL/6j and C3H/HeJ*Pde6b*^*rd1*^ mice were purchased from the Jackson Laboratory (Bar Harbor, ME) and bred in the animal facility of Penn State College of Medicine. Mice were housed in standard cages with a 12h light/12h dark cycle. Rodent chow and water were provided ad libitum.

### Genome-wide databases

Data sets were collected from the NCBI GEO data repository and visualized in the UCSC Genome Browser (www.genome.ucsc.edu). Data sets for H3K4me2 CHIP-Seq were from mouse retina at E17, PN1 PN7, PN15 of wild type (WT) mice and from retina of mutant RD1 PN30 (GSE38500, [31]); mouse early and late erythroblast cells from embryonic liver (GSE27893, [36]); mouse NP (neuronal progenitor) and WB (whole brain) (GSE11172, [37]).

For tag distribution around TSS we used RefSeq database of established transcription start sites [38] and the number of reads were calculated in the window TSS+/- 1000bp.

The CRX–binding site ChIP-Seq data set was from WT retina at PN56 (GSE20012, [33]). The number of reads was calculated in a window of +/- 1000bp around the TSS. We classified TSS according to CRX binding at the cluster of genes of interest. Binding was clearly bimodal with minimum binding more than 300 reads, 0 reads was classified as no binding and everything above this as positive binding (Fig A in S1 File).

Similarly, we studied NRL binding to TSS using a published Chip-Seq data set from WT retina PN28 [32]. The number of reads was calculated in a window of +/- 1000bp around the TSS. Binding was clearly bimodal and TSS with 0 reads was classified as no binding and everything above this as positive binding (Fig B in S1 File).

The ChIP-on-Chip dataset for Polymerase Pol-II binding sites was obtained from WT retina at PN2 and PN25 (GSE19999, [34]). We estimated stage-specific PolII occupancy in +/-1000bp window of each TSS for genes in S1 Table. We then calculated level of PolII-binding changes during development by subtracting PolII occupancy at PN2 from PolII occupancy at PN25 (ΔPolII). We divided TSS accordingly to developmental changes in PolII binding in three groups: PolII is not binding to TSS (ΔPolII = 0), PolII binding is low but up- regulated during development (0 < ΔPolII < 182, where 182 = two S.D. + mean), PolII binding is highly up-regulated during development (ΔPolII>182).

Mouse DNase1 Hyper Sensitive sites were collected from the ENCODE project: mouse retina PN1(1D), PN7 (1W), PN56 (8W) (GSM1014188; GSM1014198; GSM1014175)[35]; mouse whole brain from E14.5, E18.5 and PN56 (GSM1014197, GSM1014184, GSM1014151). Each dataset above was intersected with each isoform’s TSS +/- 1000 bp, with the score contribution of a dataset entry being set to the number of intersecting bps times the score of the entry. We calculated ratio of DHS changes during development by dividing of DHS occupancy at PN56 and PN1 (PN56/PN1). We then divided TSS according to developmental changes in DHS occupancy in four groups: DHS is down regulated during development (DHS PN56/PN1 < 1), DHS is up regulated during development (1< DHS PN56/PN1 <1.35), DHS is strongly up regulated during development (DHS PN56/PN1 > 1.35) (where 1.35 = two S.D+ mean).

Developmental profiles of gene expression were from reanalyzed data of microarrays previously described [16]. Expression was normalized to a pool of equal amounts of all seven ages tested, resulting in a maximum sevenfold change in expression.

### Data access

We have made data viewable on UCSC mouse Genome Browser mm9 with our data sets for H3K4me2/ H3K27me3 profiles, as well publicly available data sets for DHS (ENCODE project, [35]), PolII- and CRX binding sites [33, 34] (Barnstable lab retina epigenome)

### Cluster analysis

Hierarchical cluster analysis was performed with Gene Cluster 3.0[39] and Java Tree View was used for clusters visualization or with the heatmap.2 function of the gplots R package, using (1-correlation)/2 as the distance and average as the clustering method. The Z scaling was obtained from the genefilter v1.46.1 R package.

For cluster comparisons we use IBM developed analysis for clusters (https://www.ibm.com/support/knowledgecenter/SSLVMB_20.0.0/com.ibm.spss.statistics.help/alg_cluster-evaluation_goodness.htm). In particular, the ‘silhouette coefficient’ combines the concepts of cluster cohesion (favoring models which contain tightly cohesive clusters) and cluster separation (favoring models which contain highly separated clusters). It can be used to evaluate individual objects, clusters, and models. Average Silhouette coefficient: (B-A)/max(A,B) where A is the average distance from the case to every other case assigned to the same cluster and B is the minimal average distance from the case to cases of a different cluster across all clusters. An average silhouette coefficient greater than 0.5 indicates reasonable partitioning of data while average silhouette coefficient less than 0.2 means that the data do not exhibit cluster structure.

### Tissue collection

Animals were euthanized in Euthanex Auto CO_2_ System (www.euthanex.com). Whole retinas were isolated from animals by quickly removing the sclera and most of the retinal pigmented epithelium (RPE) layer in PBS. Spleen, brain, lung and liver tissue were quickly isolated from mouse and cleaned of connective tissue. Immediately after isolation, tissue was flash frozen in liquid nitrogen and stored in -80°C. 30 mg of tissue or 2 retinas were used for each RNA extraction.

### RNA extraction and cDNA preparation

RNA extraction and purification followed the manufacturer’s protocol from RNeasy Mini Kit and RNA shredder (Qiagen). Buffer RLT was added to the frozen tissue and spleen, brain, lung or liver tissue was disrupted in a teflon-glass homogenizer and retinas were homogenized by pipet trituration at room temperature according to the Kit protocol. Final RNA concentrations were determined spectrophotometrically using a GeneSpect III (Hitachi Tokyo, Japan). cDNA was synthesized with SuperScriptII First-Strand Synthesis System kit according to manufacturer’s protocol (Invitrogen, Carlsbad, California).

### RT-PCR

Primers were designed and purchased from Integrated DNA Technologies (IDT). The sequence information listed in S1 Table. For quantitative real-time PCR we used 2x iQ-SYBR Green PCR supermix from Bio-Rad. Samples in triplicate were run on iQ5 Multicolor Real Time PCR Detection System (Bio-Rad).

### Statistical analysis

Statistical analyses were performed using the Excel or GraphPad Prism software. Student’s t-test (two-tailed, unpaired) was used to compare two groups.

## Results

### Epigenetic signatures indicate tissue-specific use of TSSs of genes expressed in many tissues

Among the 107 genes with a distinct rod photoreceptor epigenetic signature identified in our previous study were a group of 30 with a single TSS that have previously been shown to be expressed exclusively in rod photoreceptors [31]. Throughout this earlier study, this set of 30 known rod genes was used as a training set to help define the characteristics of other transcripts selectively expressed in rod photoreceptors. A wide range of databases and publications have documented that 102 of the 107 genes identified in our earlier study show expression in the eye; there is no published information on the other 5. In addition to the published data, a group chosen at random showed an increase in retinal expression that matched the time course of rod differentiation, and decreased expression in the RD1 mutant at a time when all the rods have degenerated [31], thus supporting the idea that these genes are all expressed in rod photoreceptors.

**Fig 1:**
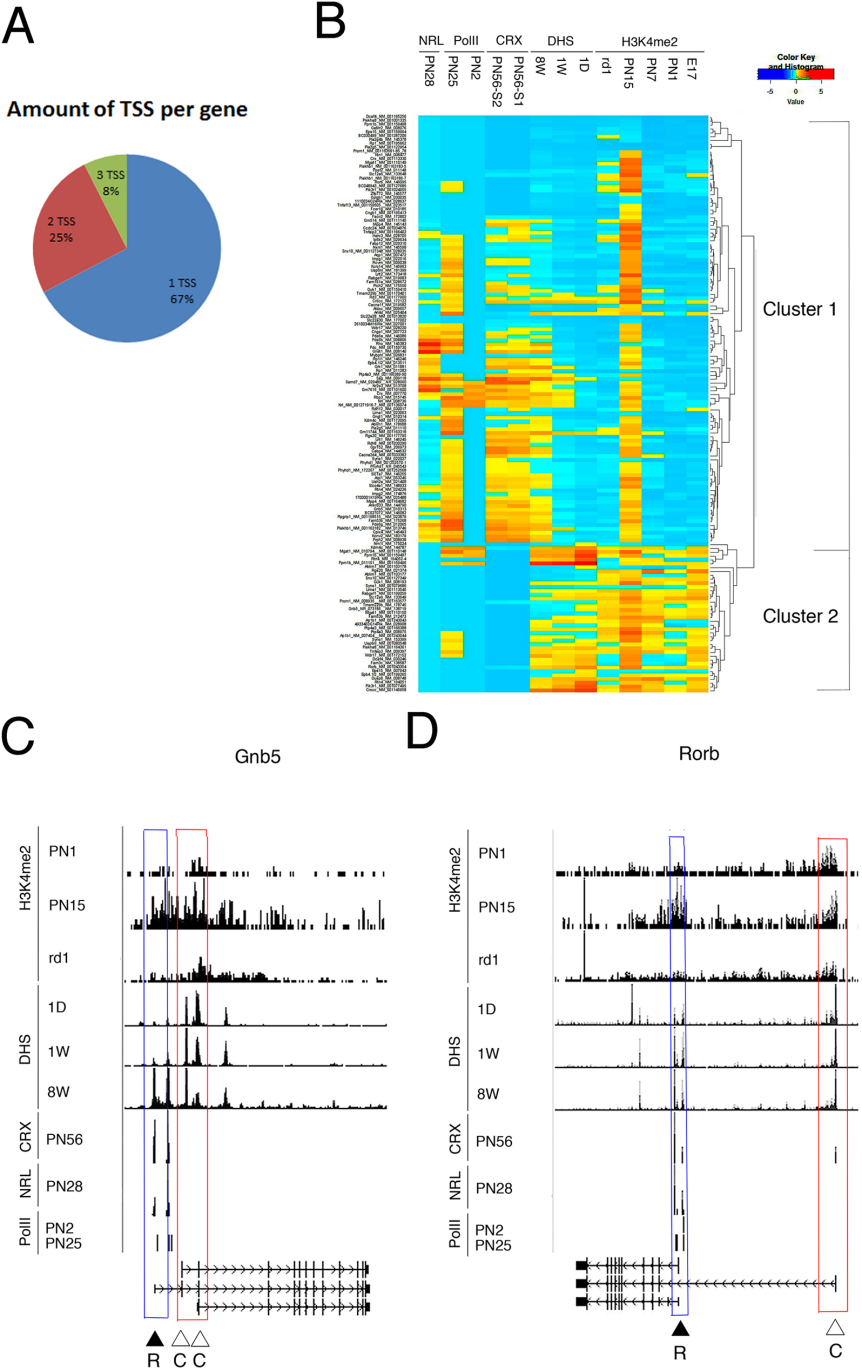
Epigenetic signature predicts employment of ATSS of ubiquitous gene in tissue-specific manner. **A.** Pie chart presentation for number of TSS for different groups of rod-specific genes. **B.** All TSS for rod-specific genes were clustered based on chromatin signature around TSS+/-1000bp for following features: developmental changes in H3K4me2 occupancy (E17, PN1, PN7, PN15 and RD1 PN30), CRX-binding (PN56), NRL-binding (PN28), developmental changes in DHS (1D, 1W, 8W), developmental changes in PolII (PN2, PN25) as clustering criteria (see methods for details). **C, D.** Combined genome-wide tracks of chromatin features, as in **B** for *Gnb5* and *Rorb* genes. Common TSS is depicted as C/ red box, rod TSS–as R/ blue box.

Of the 107 genes with a rod specific epigenetic signature, 72 (67%) have a single identified TSS listed in the RefSeq database. Of the other 35 genes, 27 have two identified TSS and 8 have 3 TSS, for a total of 78 TSS (Fig 1A, S2 Table). These alternative TSSs lead 25 of the genes to produce different isoforms of the protein and 10 of the genes to produce different mRNAs but the same protein isoform (S2 Table).

To explore further the relationship of the epigenetic signatures around TSS (+/-1000bp) with other characteristic markers of gene activity we extracted data for several chromatin and transcriptional features for the 107 genes defined by all their TSS. In addition to H3K4me2 occupancy (at ages E17, PN1, PN7, PN15 and RD1), we used data on binding of the two best characterized rod photoreceptor transcription factors CRX (at age PN56) and NRL (at age PN28). We also used the developmental appearance of DHS (at ages PN1(1D), PN7(1W), PN56(8W)) and the general transcription marker of PolII-binding (at ages PN2 and PN25) (S3 Table). For each gene discussed below we provide all the marker data but discuss the general applicability of each marker in separate sections below. RD1 mouse PN30 retina, an age where the retina has lost all rod photoreceptors, was used as negative control. Bipolar cells undergo extensive remodeling during neurodegeneration and in RD1 mice (review in[40]), but expression of rod-specific genes as Rho, Sag and Rorb show substantial down regulation in gene expression arrays that compare mouse retina at 3 developmental points from RD1 mutant and wild type retina [41].

We first performed a hierarchical clustering analysis using genome-wide data for all of these markers available for mouse retina (Fig 1B). All TSS fell into 1 of 2 very distinct clusters. Cluster 1 contains the TSS from all 72 genes with a single TSS, including all of our training set of known rod genes, and one (or more) TSS of the genes with multiple TSS (S4 Table). Cluster 1 TSS can be defined by the combination of H3K4me2 accumulation during development (less H3K4me2 in retina of RD1 mice), PolII-binding and DHS increase later in development and binding of CRX and NRL. We refer to these as *rod TSS* throughout this paper.

Cluster 2 defines the additional TSS from the 35 genes with multiple TSS (S4 Table). These TSS show a constant accumulation of H3K4me2 and constant DHS during development, as well as no NRL or CRX-binding, suggesting that they could be used constitutively in retina and possible, other cell types. We refer to these as *common TSS* throughout this paper.

Rods are already postmitotic at PN7, but transcription of the large group of later-expressed true rod-specific gene (like Rho, Pde6b) starts at PN7 and then dramatically increases in later stages from PN15 to adulthood (see Fig 1A in [31] and Fig 2D in [16]). Because of this, neither H3K4me2, nor DHS at PN7 show big accumulation at TSS for well-established rod-specific genes. The same is true for our newly predicted rod TSS. As epigenetic profiles for later-expressed genes are very similar at PN1 and E17, and PN7 represented a stage of transition, to simplify figures and further analysis we used PN1 comparing it with PN15 for H3K4me2, and PN1 (1D) and PN56 (8W) for DHS.

To test whether the different TSS environments reflect cell type and temporal specificity we examined two well-characterized rod genes, *Gnb5* and *Rorb*, both exist as two protein isoforms where the tissue expression of each is known (Fig 1C and 1D). *Gnb5* is a member of the G-protein beta subunit family. GNB5 expression is found almost exclusively in the nervous system. The longer form is expressed only in the retina [42], is important for regulating G protein-mediated signaling cascades in photoreception [43], and its TSS (depicted as R in Fig 1D) has the features of the rod epigenetic signature. The shorter protein form of GNB5 is expressed in brain and brain-derived cell lines [44, 45] and its two TSS (depicted as C in Fig 1D) show unaltered high H3K4me2 accumulation for all developmental stages. These two common TSS are marked by DHS regions present at birth but the rod TSS develops a DHS in parallel with the time course of rod development. The rod TSS also shows CRX and NRL, binding features absent in the common TSS.

The *Rorb* gene has 2 protein isoforms, transcribed from two TSS where the first exons differ between the two forms. The longer form, RORβ1 is expressed broadly in the different parts of the brain [46] and amacrine and horizontal cells early in retina development [47]. Its common TSS (depicted as C in Fig 1D), has high levels of H3K4me2 throughout development, no change in H3K4me2 accumulation in the RD1 mutant, a constant DHS even at PN1, and no NRL binding. The shorter form, RORβ2 is expressed later during retina development and is important for rod differentiation [48]. Its rod TSS (depicted as R in Fig 1D), on the other hand has the rod epigenetic signature, a DHS that develops postnatally and both CRX and NRL binding in TSS+/- 1000bp window that is coincident with the DHS. For both *Gnb5* and *Rorb* the known cell type expression of the protein isoforms can successfully be predicted by the TSS structure.

**Fig 2:**
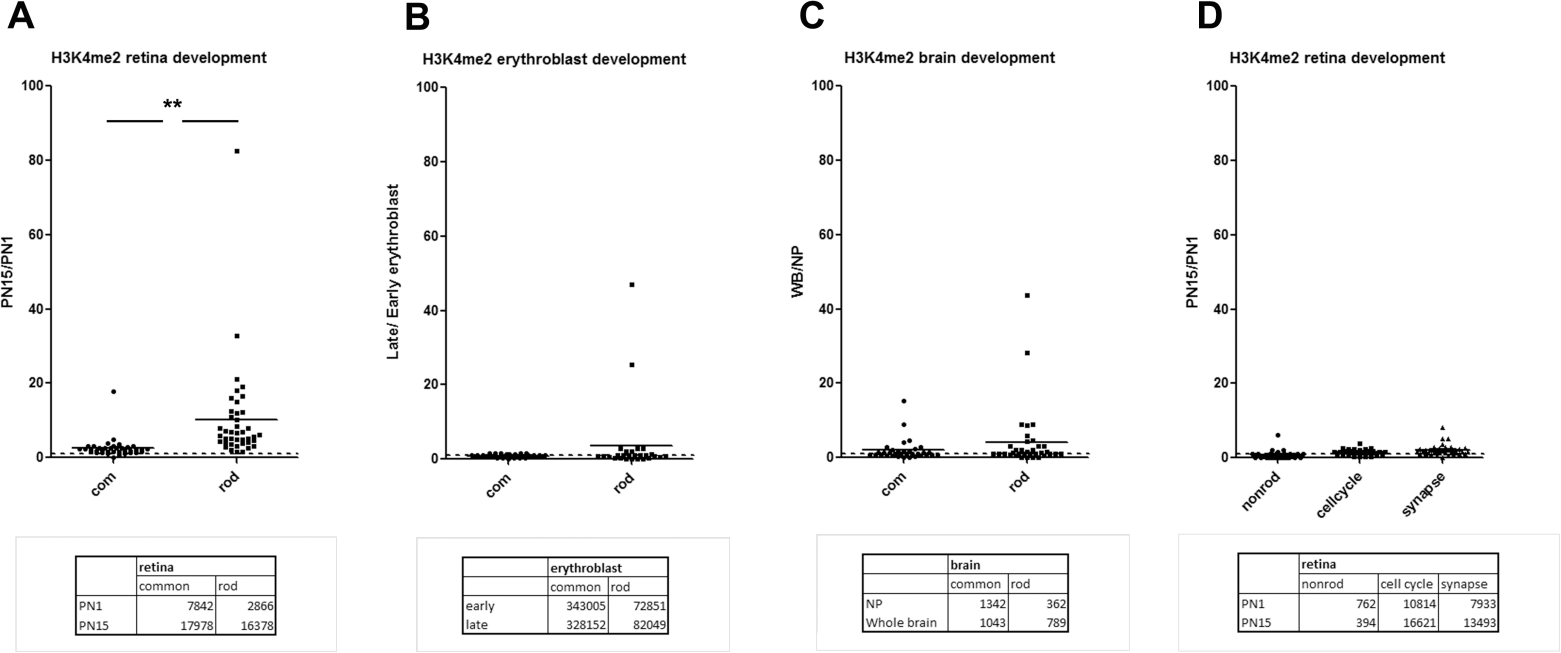
H3K4me2 epigenetic signature predicts tissue and cell specificity of TSS. **A.** Changes of H3K4me2 occupancy around TSS+/-1000bp during mouse retina development as ratio of normalized number of reads at PN15/PN1 for rod and common TSS of retina genes with multiple TSS. At the bottom: average number of H3K4me2 reads accumulation for each group of TSS at PN1 and PN15. **p = 0.0011. **B.** Changes of H3K4me2 occupancy around TSS+/-1000bp during mouse erythropoiesis as ratio of normalized number of reads between late and early erythroblast for rod and common TSS of retina genes with multiple TSS. At the bottom: average number of H3K4me2 reads accumulation for both groups of TSS at early and late stages. **C.** Changes of H3K4me2 occupancy around TSS+/-1000bp during mouse brain development as ratio of normalized number of reads for whole brain and neuronal progenitor for rod and common TSS of retina genes with multiple TSS. At the bottom: average number of H3K4me2 reads accumulation for both groups of TSS at early and late stages. **D.** Changes of H3K4me2 occupancy around TSS+/-1000bp during mouse retina development for TSS of control groups of genes (non-rod, cell-cycle and synapse).

We next explored the epigenetic signatures around the TSSs of the set of the 35 genes with multiple TSS. During retina development, the TSSs that we predict define a rod transcript accumulate the active epigenetic mark H3K4me2 with the mean PN15/PN1 ratio of 10.1+/-2.1 in the region +/- 1000bp around the TSS (Fig 2A). The other TSSs, that we predict produces a tissue common transcript, did not show such a large change in accumulation and had a mean ratio of 2.6+/-0.5. The 4 fold higher ratio in the rod TSSs was highly significant (p = 0.0011). We then examined H3K4me2 accumulation at the TSS of the same genes in blood between late and early erythroblast [36] and for brain between whole brain (WB) and neuronal progenitor (NP) [37]. There were no significant developmental differences in H3K4me2 accumulation for either rod or common TSS of the 35 genes during blood (Fig 2B) or brain (Fig 2C) development.

To exclude the possibility that the developmental increase in H3K4me2 at rod TSSs might be a general developmental effect rather than a rod specific effect, we picked approximately 30 genes for three different functional groups: cell cycle and proliferation genes, genes involved in neural processes and synapses, and genes not expressed in retina (S5 Table). None of these genes showed any significant developmental difference in accumulation on H3K4me2 around TSS in retina (Fig 2D).

These data support the idea that for genes that may be expressed in multiple tissues, alternative TSS can be used to generate tissue-specific transcripts and can be recognized by their epigenetic signature.

### Biological confirmation of relationship between epigenetic signatures and gene expression

**Fig 3:**
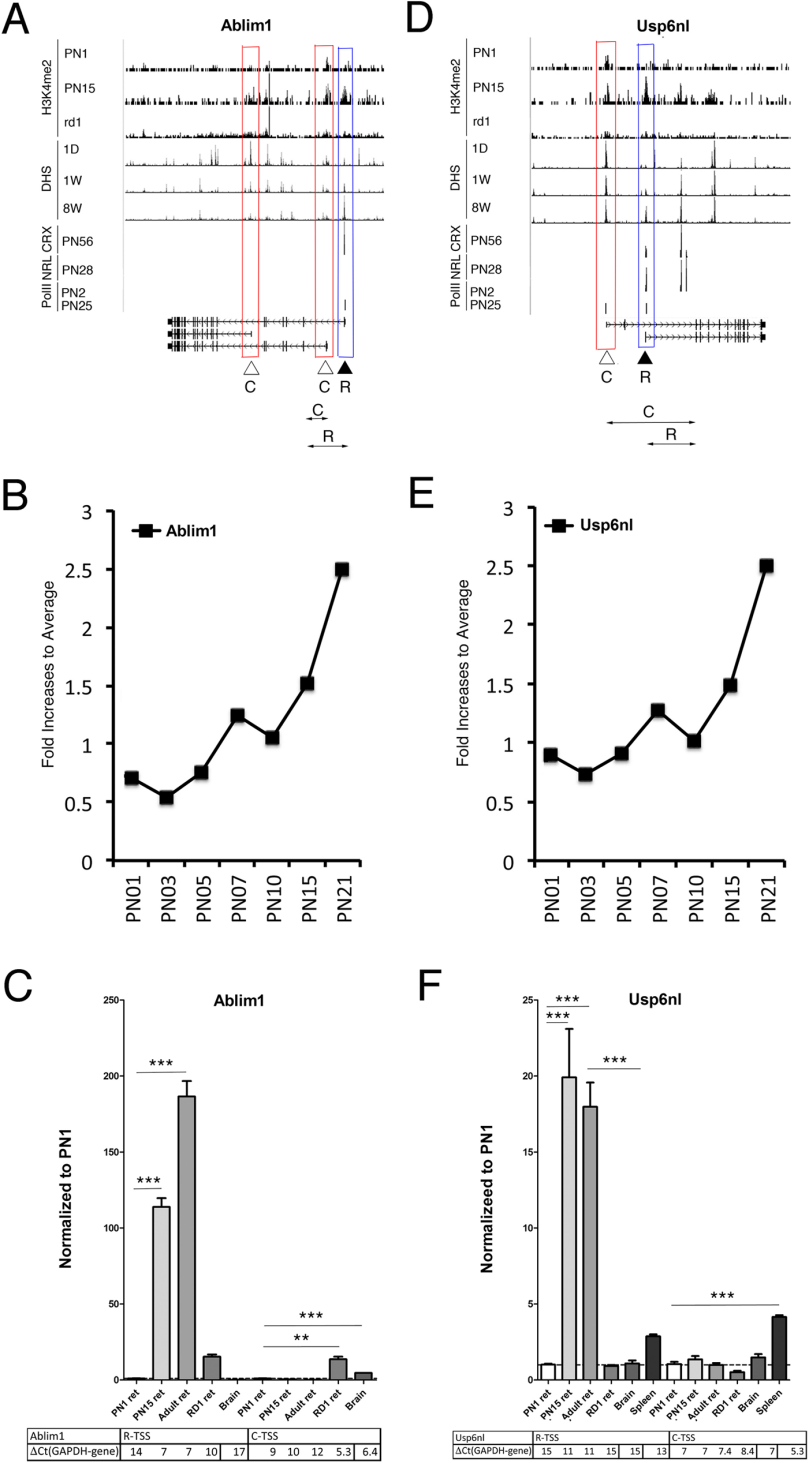
Confirmation of relationship between epigenetic signatures and gene expression. **A, D.** Combined genome-wide tracks of chromatin features, as in **Fig 1B** for *Ablim1* (**A**) and *Usp6nl* (**D**) genes. Common TSS is depicted as C/ red box, rod TSS–as R/ blue box. Position of the specific primer sets and PCR product that were used to assess and distinguish TSS-specific gene expression by RT-PCR depicted as double arrow below gene maps. **B, E.** Patterns of *Ablim1* and *Usp6nl* genes expression during mouse retina development; reanalyzing of microarray data from[16]. **C, F.** Relative gene expressions from rod and common TSS by RT-PCR with primer pairs depicted at **A and D** for *Ablim1* (**C**) and *Usp6nl* (**F**) for mouse retina samples at PN1, PN15, adult and RD1 mutant, compare with mouse brain and spleen. For comparison, normalized to *Gapdh* delta Ct values for each sample are in table below. Experiments done in duplicates with three technical replicas; ***—p < 0.0001.

We compared levels of expression from the rod and common TSS of several of the genes with alternative TSS (S2 Table) using qRT-PCR with primers designed to identify the different transcripts from each TSS. Two examples are shown in Fig 3.

The *Ablim1* gene has three alternative TSS with all forms having different first exons and different protein sequences (Fig 3A). *Ablim1* transcripts have a widespread tissue distribution and microarray data demonstrate an overall increase in expression during retina maturation (Fig 3B); however, the longest isoform is found exclusively in retina [16, 49]. This longest isoform has all the characteristics of being transcribed from a rod TSS. The other two TSS show a similar H3K4me2 distribution between PN1 and PN15, little change in DHS and no CRX or NRL binding (Fig 3A). We confirmed by RT-PCR that the proposed rod isoform is expressed primarily in postnatal retina, while one of transcripts from common TSS has higher expression in the RD1 mutant that has lost rods (Fig 3C).

The *Usp6nl* gene encodes a GTPase activator that is required for the structural integrity of the Golgi complex. It has two alternative TSS and each form has a different first exon and encodes a different protein (Fig 3D). Previously published expression array data [16] demonstrate an overall increase in *Usp6nl* expression during retina maturation (Fig 3E). Inspection of the TSS regions shows that one of the TSS has the properties of a rod TSS and the other of a common TSS (Fig 3D). RT-PCR amplification with specific primer sets confirmed that the transcript from the rod TSS generates an isoform expressed mostly later in postnatal retina and lost in the RD1 retina, while the other TSS generates a transcript, that shows no difference in expression during retina development and in RD1 (Fig 3F).

**Fig 4:**
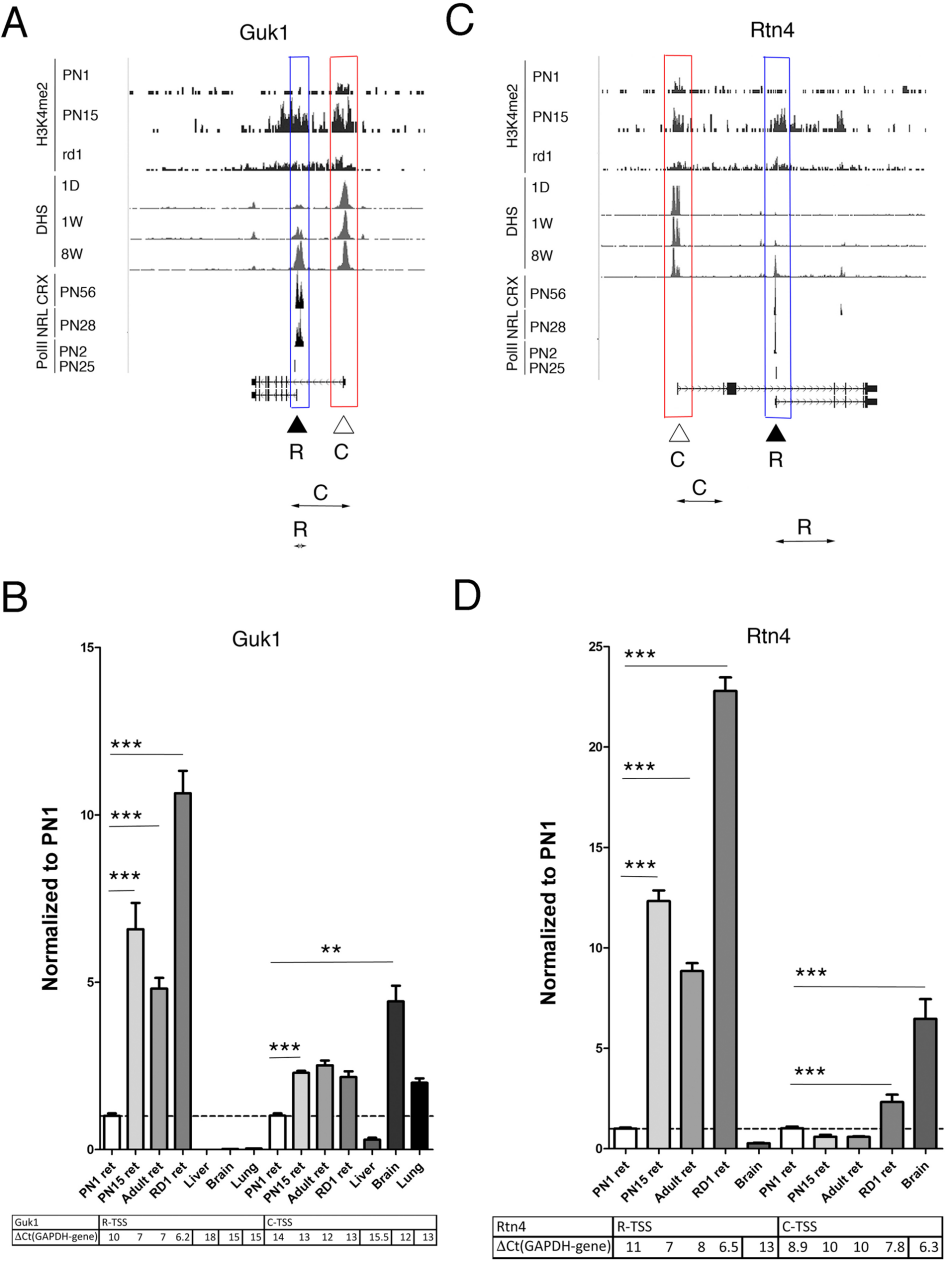
Examples of the genes that use alternative transcription start sites in retina. **A, C.** Combined genome-wide tracks of chromatin features, as in **Fig 1B** for *Guk1* (**A**) and *Rtn4* (**C**) genes. Common TSS is depicted as C/ red box, rod TSS–as R/ blue box. Position of the specific primer sets and PCR product that were used to assess and distinguish TSS-specific gene expression by RT-PCR depicted as double arrow below gene maps. **B, D.** Relative gene expressions from rod and common TSS by RT-PCR with primer pairs depicted at **A and C** for *Guk1* (**B**) and *Rtn4* (**F**) for mouse retina samples at PN1, PN15, adult and RD1 mutant, compare with mouse brain, liver and lung. For comparison, normalized to *Gapdh* delta Ct values for each sample are in table below. Experiments done in duplicates with three technical replicas; **—p <0.001; ***—p < 0.0001.

Only two of our set of new rod genes, *Guk1* and *Rtn4*, gave anomalous results (Fig 4). Although the epigenetic signature, DHS accumulation, both CRX and NRL binding profiles predict rod specificity for one of the TSS and mRNA for the apparent rod specific form increased during postnatal development, expression was much higher in adult RD1 retinas. Neither TSS properties nor expression of *Guk1* and *Rtn4* genes from the other TSS (Fig 4) were altered during development, as expected from a tissue common TSS.

### Association of rod photoreceptor transcription factors CRX and NRL with rod TSS

To examine the reliability of using transcription factors to define cell type specific TSS, we combined our genome-wide chromatin signature maps with the whole-genome ChIP-Seq data for CRX and NRL [32, 33]. We calculated CRX and NRL accumulation in +/- 1000bp window of each TSS for all 107 genes.

**Fig 5:**
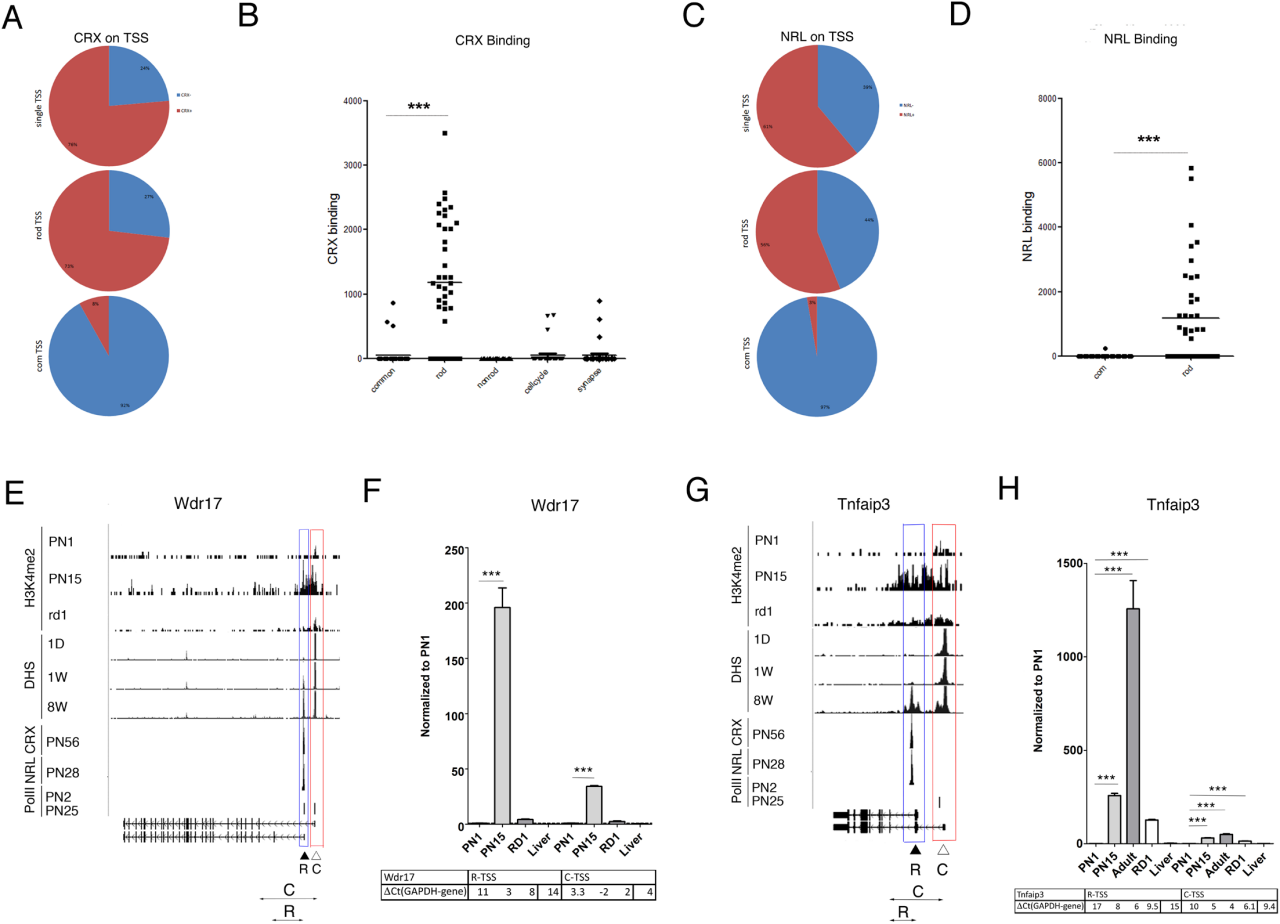
Association of rod photoreceptor transcription factors CRX and NRL with rod TSS. **A, C.** Number of TSS with different CRX (**A**) and NRL (**C**) binding for rod-specific genes with single TSS (upper panel) and for rod (middle panel) or common (bottom panel) TSS of retina genes with multiple TSS. Blue–no TF-binding; red–TF-binding. **B, D.** CRX (**B**) and NRL (**D**) accumulation at TSS (normalized number of reads at TSS+/-1000bp) for rod and common TSS of retina genes with multiple TSS, compared with control groups of genes (non-rod, cell-cycle and synapse). ***—p < 0.0001. **E, G.** Combined genome-wide tracks of chromatin features, as in Fig **1B** for *Wdr17* (**E**) and *Tnfaip3* (**G**) genes. Common TSS is depicted as C/ red box, rod TSS–as R/ blue box. Position of the specific primer sets and PCR product that were used to assess and distinguish TSS-specific gene expression by RT-PCR depicted as double arrow below gene maps. **F, H.** Relative gene expressions from rod and common TSS by RT-PCR with primer pairs depicted at **E and G** for *Wdr17* (**F**) and *Tnfaip3* (**H**) for mouse retina samples at PN1, PN15, adult and RD1 mutant, compare with mouse liver. For comparison, normalized to *Gapdh* delta Ct values for each sample are in table below. Experiments done in duplicates with three technical replicas; ***—p < 0.0001.

Since the amount of CRX binding showed a clear bimodal distribution (Fig A in S1 File), we classified genes as having or not having CRX binding in the vicinity of their TSS. Of the 72 genes with a single TSS 55 (76%) had clear CRX binding (Fig 5A). Of the 35 genes with multiple TSS, 41 TSS matched the rod profile (cluster 1 in Fig 1A) and of these 30 (73%) had clear CRX binding (Fig 5A) similar to rod genes with single TSS. In contrast, the 37 TSS defined as common from the epigenetic profiles, only 8% showed CRX binding. The 11 (27%) rod TSS that appeared to have no CRX binding were examined in more detail. Five of these (*Mgat1*, *Ap1b1*, *Pik3r1*, *Plekha8*, *and Prom1*) did have CRX binding but more than 1000 bp from the TSS.

When the levels of CRX binding were plotted, the mean level for the rod TSS was 22 times that for the common TSS (Fig 5B). Analysis of the groups of control non-rod, cell cycle and synapse genes as used earlier showed that their TSS demonstrated almost no CRX binding.

We next analyzed NRL binding to TSS in the same way. This too was bimodal (Fig B in S1 File) and we were able to define genes as having or not having NRL binding. Of the 72 genes with a single TSS 44 (61%) had NRL binding (Fig 5C). Of the 35 genes with multiple TSS, 23 (56%) of the 41 rod TSS showed substantial NRL binding, similar to rod genes with single TSS, but only 1 (3%) of the 37 common TSS showed NRL binding (Fig 5C). When the levels of NRL binding were plotted for each TSS, the average for the rod TSS was 172 times greater than for the common TSS (Fig 5D). The TSS of the three control groups of genes had no NRL binding above threshold (data not shown).

Genome–wide tracks for two genes, *Wdr17* (Fig 5E) and *Tnfaip3* (Fig 5G), exemplify these patterns of retinal TF binding at rod TSS. *Wdr17* is a candidate for the RP29 form of Retinitis Pigmentosa [50, 51] and has binding sites for CRX and NRL close to the rod TSS. The common TSS directs expression in brain, retina and testis and lacks both CRX and NRL binding (UCSC EST database[51]). Because the two transcripts differ in their first exons we were able to confirm by RT-PCR that the transcript from the rod TSS is upregulated ~200 times in postnatal retina, while the transcript from common TSS shows a smaller, though still significant increase (Fig 5F). We concluded that, in retina, rod photoreceptor transcription factors CRX and in less extent NRL are associated with rod TSS for genes with multiple TSS.

*Tnfaip3* has been identified as a negative regulator of the NF-κB pathway and has not previously been implicated in retina development or maintenance. One TSS of this gene demonstrates features of expression in rods namely CRX and NRL binding, as well as H3K4me2 accumulation later in development (Fig 5G). For the rod TSS we confirmed by RT-PCR that its transcript is upregulated over a thousand fold in postnatal retina, while the transcript from common TSS had a much smaller change in expression between PN1 and adult. (Fig 5H).

### Rod TSSs are associated with DNase1 hypersensitive sites in retina

**Fig 6:**
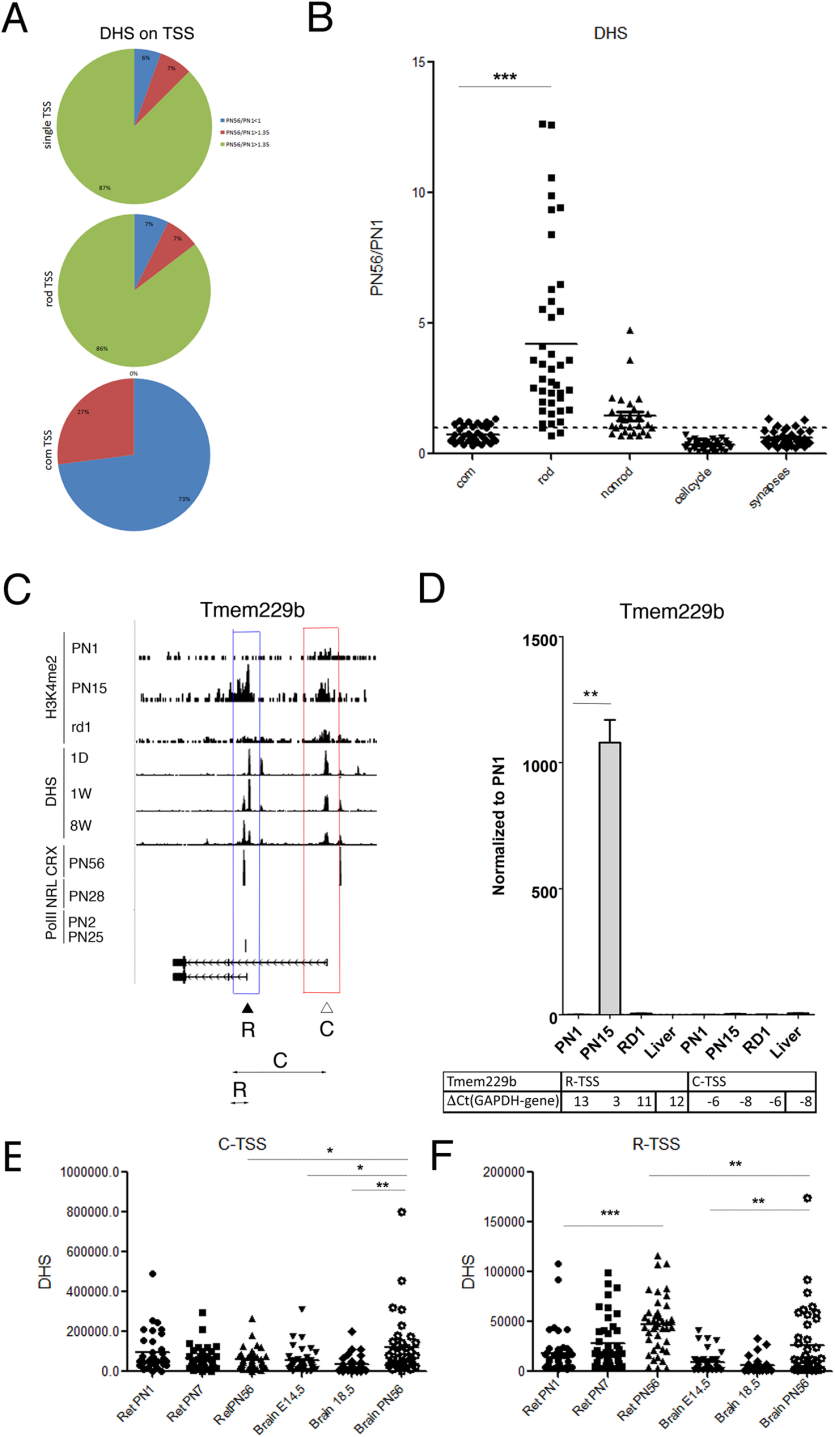
Rod TSSs are associated with DNase1 hypersensitive sites in retina. **A.** Number of TSS with different ratio of DHS changes during retina development (comparison of DHS occupancy at PN56 and PN1; PN56/PN1) for rod genes with single TSS (upper panel) and for rod (middle panel) or common (bottom panel) TSS of retina genes with multiple TSS. Blue–no changes; red–low PN56/PN1; green- high PN56/PN1. **B.** Developmental changes in accessibility of different TSS by DHS (PN56/PN1 DHS at TSS+/-1000bp) for rod and common TSS of retina genes with multiple TSS, compared with control groups of genes (non-rod, cell-cycle and synapse). ***—p < 0.0001. **C.** Combine genome-wide tracks of chromatin features, as in **Fig 1B** for *Tmem229b* gene. Common TSS is depicted as C/ red box, rod TSS–as R/ blue box. Position of the specific primer sets and PCR product that were used to assess and distinguish TSS-specific gene expression by RT-PCR depicted as double arrow below gene map. **D.** Relative gene expressions from rod and common TSS by RT-PCR with primer pairs depicted at **C** for *Tmem229b* for mouse retina samples at PN1, PN15, adult and RD1 mutant, compare with mouse liver. For comparison, normalized to *Gapdh* ΔCt values for each sample are in table below. Experiments done in duplicates with three technical replicas; **—p < 0.001. **E, F.** Accessibility of different TSS by DHS (number of DHS at TSS+/-1000bp) during mouse retina (PN1, PN7, PN56) and brain (E14.5, E18.5, PN56) developments for rod (**F**) and common TSS (**E**) of retina genes with multiple TSS.*—p = 0.026 (**E**); ***—p = 0.0003 (**F**); *—p = 0.037/ 0.01.

A catalog of retinal DNase1 Hypersensitive Sites (DHS) (University of Washington data) is available for 3 developmental stages, PN1 (1D), PN7 (1W), PN56 (8W) [35]. To compare rod-specific epigenetic signatures with DHS we monitored developmental changes in DHS for all 107 genes, estimating the stage-specific DHS occurrence in +/-1000bp window of each TSS. We calculated a ratio of DHS change during development by dividing DHS occupancy at PN56 with that at PN1 (PN56/PN1), and classified the TSS into three groups: DHS is down regulated during development, DHS is up regulated during development, or DHS is strongly up regulated during development. For the 72 rod genes with a single TSS, 94% showed a developmental increase in DHS; with 5 genes have DHS upregulated and 63 have DHS strongly upregulated (Fig 6A, upper panel). For the 41rod TSS of the 35 genes with multiple TSS, 93% showed a DHS increase similar to the single TSS rod genes; with 3 genes have DHS upregulated and 35 have DHS strongly upregulated (Fig 6A, middle panel). For the 37 common TSS of these genes, 27 (73%) demonstrated a decrease in DHS during development (Fig 6A, lower right panel). Plotting individual changes in DHS for genes with multiple TSS (Fig 6B), indicated that the PN56 to PN1 ratios of the rod TSS showed a wide variation but on average were 5 times greater than the ratios for the common TSS. Control groups of genes showed no significant change in DHS during development, similarly to common TSS.

Genome–wide tracks for one gene, *Tmem229b* (Fig 6C), illustrates the developmental increase in chromatin accessibility at a DHS for a rod TSS that lines up with CRX binding. The *Tmem229b* gene is expressed in a number of tissues and its common TSS is associated with unique ESTs from whole brain and bone marrow, while the rod TSS gives rise to only retina ESTs (UCSC EST database). For the proposed isoform generated from the rod TSS we confirmed by RT-PCR that it is upregulated a thousand fold between PN1 and PN15, while the transcript from the common TSS had constant high level of expression (Fig 6D).

To confirm tissue specificity of DHS changes for the TSSs of genes with multiple TSS we compared temporal changes in the DHS around rod and common TSS (TSS +/- 1000bp) for retina (ENCODE PN1, PN7, PN56) and brain (ENCODE E14.5, E18.5, PN56) development. For the common TSS only DHS in the brain development group showed changes (Fig 6E). For the rod TSS there was a clear increase in DHS over time in retina but much smaller change in brain (Fig 6F).

From this we concluded that developmental changes in DHS at TSSs are consistent with their epigenetic profiles and can help indicate tissue-specific use of TSSs of ubiquitously expressed genes.

### Rod TSSs are associated with PolII-binding sites in retina

As a further marker of transcriptional activity we examined PolII binding and estimated stage-specific PolII occupancy in +/-1000bp window of each TSS. We calculated the PolII-binding changes during development by subtracting PolII occupancy at PN2 from PolII occupancy at PN25 (ΔPolII). We then clustered these developmental changes in PolII binding into three groups: no (or decrease) PolII binding, PolII binding increased slightly during development, and PolII increased substantially during development.

**Fig 7:**
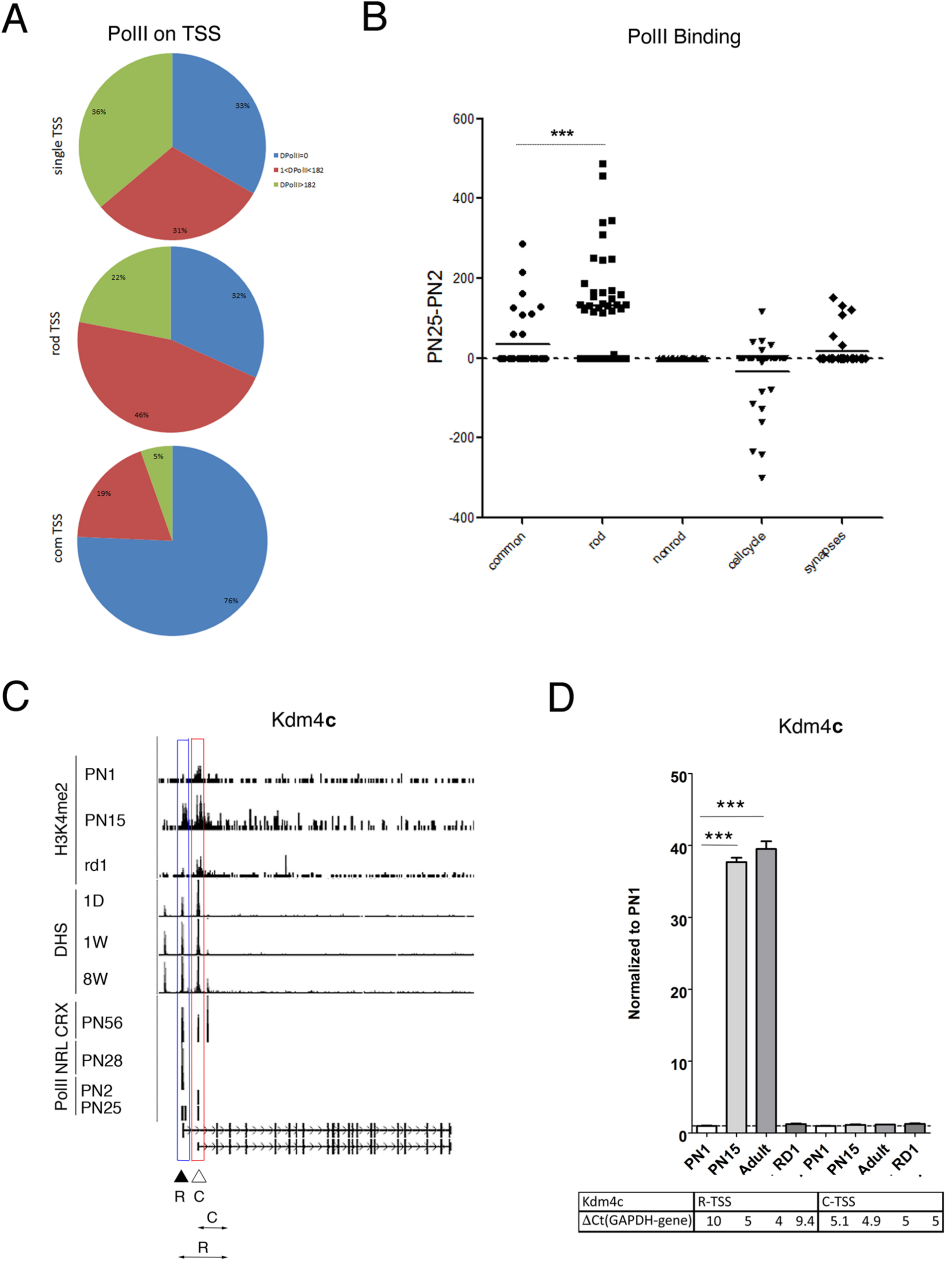
Rod TSSs are associated with PolII-binding sites in retina. **A.** Number of TSS with different ratio of PolII-binding during development (comparison of PolII occupancy at PN25 and PN2; PN25-PN2) for rod-specific genes with single TSS (upper panel) and for rod (middle panel) or common (bottom panel) TSS of retina genes with multiple TSS. Blue–no changes; red–low PN25-PN2; green- high PN25-PN2. **B.** Developmental changes in PolII-binding (PN25-PN2 at TSS+/-1000bp) for rod and common TSS of retina genes with multiple TSS, compared with PolII-binding at TSS for control groups of genes (non-rod, cell-cycle and synapse). ***—p < 0.0001. **C.** Combined genome-wide tracks of chromatin features, as in **Fig 1B** for *Kdm4c* gene. Common TSS is depicted as C/ red box, rod TSS–as R/ blue box. Position of the specific primer sets and PCR product that were used to assess and distinguish TSS-specific gene expression by RT-PCR depicted as double arrow below gene map. **D.** Relative gene expressions from rod and common TSS by RT-PCR with primer pairs depicted at **C** for *Kdm4c* for mouse retina samples at PN1, PN15, adult and RD1 mutant. For comparison, normalized to *Gapdh* ΔCt values for each sample are in table below. Experiments done in duplicates with three technical replicas; ***—p < 0.0001.

For the 72 rod genes with a single TSS, 67% showed a developmental increase in PolII binding; with 22 genes have slight and 26 have substantial increase of PolII binding (Fig 7A, upper panel). For the 41 rod TSS of the 35 genes with multiple TSS, 68% also showed a PolII binding increase similar to the single TSS rod genes; with 19 genes have slight and 9 have substantial increase of PolII binding (Fig 7A, middle panel). For the 37 common TSS, 28 (76%) have no PolII binding during development (Fig 7A, bottom panel).

We plotted the ΔPolII for each of the TSS for genes with multiple TSS and the control groups of genes (Fig 7B). Comparison between rod and common TSS shows 3.8 times an average increase in PolII-binding in vicinity of rod TSS (Fig 7B). The groups of control genes showed little change or even decreased in PolII-binding.

These changes are illustrated in the genome–wide tracks for *Kdm4c*, a histone demethylase implicated in development [52] (Fig 7C). PolII binding was detected at the rod TSS only in the adult whereas binding to the common TSS was the same at PN2 and PN25. As with many of the other genes in this class, the rod TSS had increasing levels of H3K4me2 with age and a DHS that increased in adult, while both were unchanged during development at the common TSS. Interestingly, CRX has binding sites at both Kdm4c TSSs, but NRL binds only to the rod TSS. We confirmed by RT-PCR that the proposed rod TSS generates a transcript that is upregulated ~50 fold in postnatal retina, while the transcript from the common TSS has a constant high expression level (Fig 7D).

We conclude that developmentally regulated PolII-binding correlates with the expression from rod TSS, while common TSS mostly shows no developmental changes in PolII binding.

### Using chromatin features to predict tissue specific new TSS

**Fig 8:**
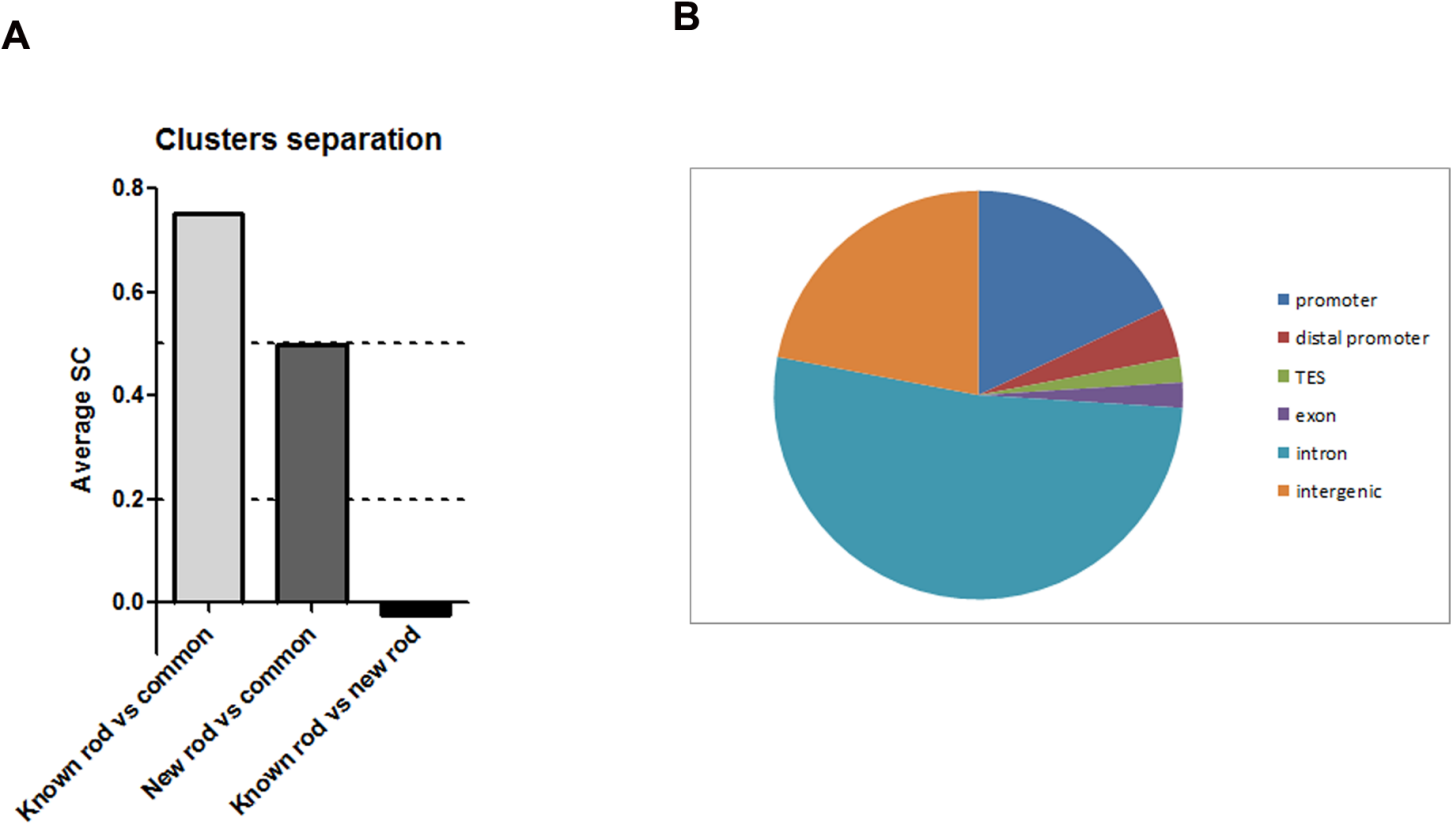
Predictive power of epigenetic profiles. A. Cluster separation for different TSS groups. SC—Silhouette coefficient. **B**. Pie chart representation of location in the genome sites that have chromatin signature characteristic for rod TSS.

To examine the predictive power of our approach we compared three clusters: TSS for known rod genes, common TSS and newly predicted rod TSS. Each TSS was characterized by 13 chromatin features (as on Fig 1B), such as H3K4me2 accumulation at E17, PN1, PN7, PN15 and in RD1; DNAse hypersensitive sites at 1D, 1W, W8; binding of PolII at PN2 and PN25; binding of CRX (samples S1 and S2) and NRL. To evaluate our clusters we used the Silhouette coefficient (SC), which combines the concepts of cluster cohesion and cluster separation. An average SC greater than 0.5 indicates reasonable partitioning of data while average SC less than 0.2 means that data do not exhibit cluster structure. Comparison between clusters of TSS for known rod genes with common TSS gave an average SC = 0.75; and comparison between clusters of newly predicted rod TSS and common TSS gave an average SC = 0.5 (Fig 8A). These data demonstrate that common TSS is a reasonable cluster and it is separated from known and newly predicted rod TSS clusters. Comparison between known and newly predicted rod TSS clusters gave an average SC = -0.02, indicating that no separation exists between these two clusters or that all these TSS belong to the same cluster, rod TSS. Thus we have been able to recognize rod TSS with a high degree of certainty.

We then tested whether our approach also had predictive power and could identify new rod TSS that were not in the current TSS database for RefSeq. Our preliminary data indicated that there are around 600 sites on mouse genome that exhibit a chromatin signature characteristic for rod TSS and 12% of these sites are located in gene loci whose human homologs are implicated in human retinal diseases in RetNet. 22% of these 600 sites are located in promoters, 2% are in exons, 2% are in Transcription End Site (TES), 22% are in the intergenic arias of the genome and 52% are in the introns (Fig 8B). Intergenic sites could represent such chromatin regulatory sites, as enhancers or insulators, while sites located in introns could be potential new rod TSS.

**Fig 9:**
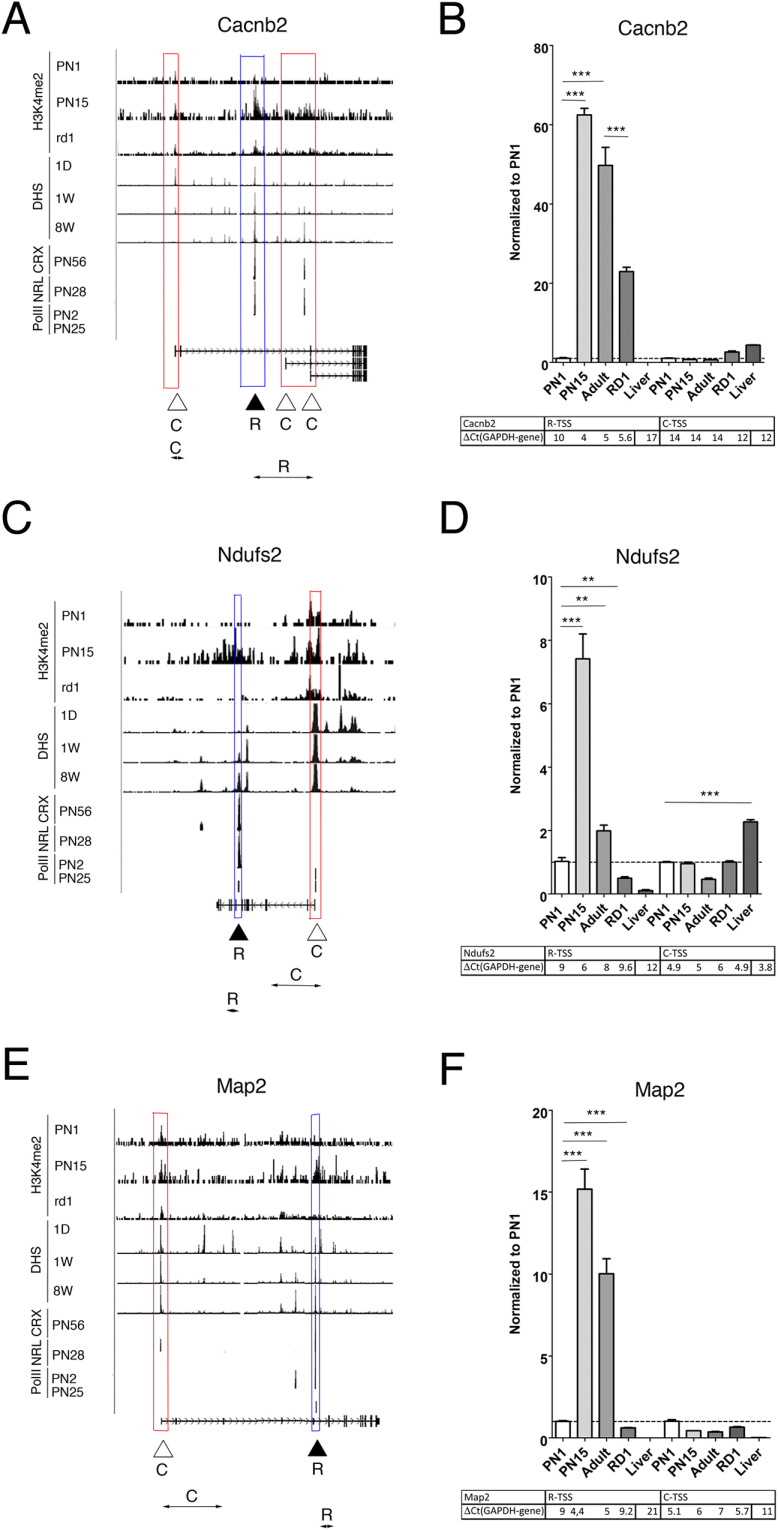
Chromatin features predict tissue specific new TSS. **A, C, E.** Combined genome-wide tracks of chromatin features, as in **Fig 1B** for *Cacnb2* (**A**), *Ndufs2* (**C**) and *Map2* (**E**) genes. Common TSS is depicted as C/ red box, predicted rod TSS–as R/ blue box. Position of the specific primer sets and PCR product that were used to assess and distinguish TSS-specific gene expression by RT-PCR depicted as double arrow below gene maps. **B, D, F.** Relative gene expressions from predicted rod and common TSS by RT-PCR with primer pairs depicted at **A, C, E** for *Cacnb2* (**B**), *Ndufs2* (**D**) and *Map2* (**F**) for mouse retina samples at PN1, PN15, adult and RD1 mutant, compare with mouse liver. For comparison, normalized to *Gapdh* ΔCt values for each sample are in table below. Experiments done in duplicates with three technical replicas; ***—p < 0.0001, **—p < 0.001.

On mouse chromosomes 1 and 2 we found 80 loci with rod TSS features, we then compared expression from this new predicted rod TSS with expression from common known TSS for four of these loci. For three out of four loci we have confirmed rod specificity for newly predicted sites. Fig 9 presents examples of the prediction of new rod TSS.

*Cacnb2*, encoding one of the beta subunits of Ca++ voltage-gated channels, is widely expressed in different tissues [53, 54]. Based on chromatin features around the gene locus we predicted that *Cacnb2* has an additional rod TSS (Fig 9A). We designed 2 pairs of PCR primers to test expression from the newly predicted rod and the common TSS. Transcription from the rod TSS is highly upregulated in adult tissue and is less in liver or RD1 retina, while transcription from common TSS is not dependent on age and is slightly upregulated in RD1 retina in to the level in liver (Fig 9B).

*Ndufs2* encodes one subunit of NADH: ubiquinone oxidoreductase (complex I) of the mitochondrial respiratory chain [55]. The known TSS of this gene in retina has the epigenetic profile of constantly expressed gene: DHS and H3K4me2 accumulation are constant during retina development, and PolII binding is similar at PN2 and PN25 (Fig 9C). Between exon 8 and 9 we found a chromatin region highly resembling the TSS profile of a rod gene: CRX and NRL binding, PolII binding only at PN25 and DHS and H3K4me2 increased at the end of retina development (Fig 9C). We probed expression from the common TSS and this new rod TSS in cDNA from retina of different ages and compared this with cDNA from liver and RD1 mutant retina. Expression from the rod TSS was upregulated during retina maturation, but was much lower in RD1 retina and liver, while transcription from common TSS showed little change in retinal development and was higher in liver (Fig 9D).

*Map2* encodes a microtubule-associated protein and produces multiple transcripts by alternative splicing that give rise to several developmentally regulated isoforms of the Map2 protein [56]. The known TSSs of this gene in retina have the epigenetic profile of a constantly expressed gene (Fig 9E). After exon 4 we found chromatin features highly resembling the TSS profile for rod genes (Fig 9E). We probed expression from common TSS and this new rod TSS in cDNA from retinas of different ages and compared this with cDNA from liver and retina from RD1 mutant retina. Expression from the rod TSS was upregulated during retina maturation, and present only at much lower levels in RD1 retina and hardly at all in liver, while transcription from the common TSS was maintained at high levels through retinal development (Fig 9F).

**Fig 10:**
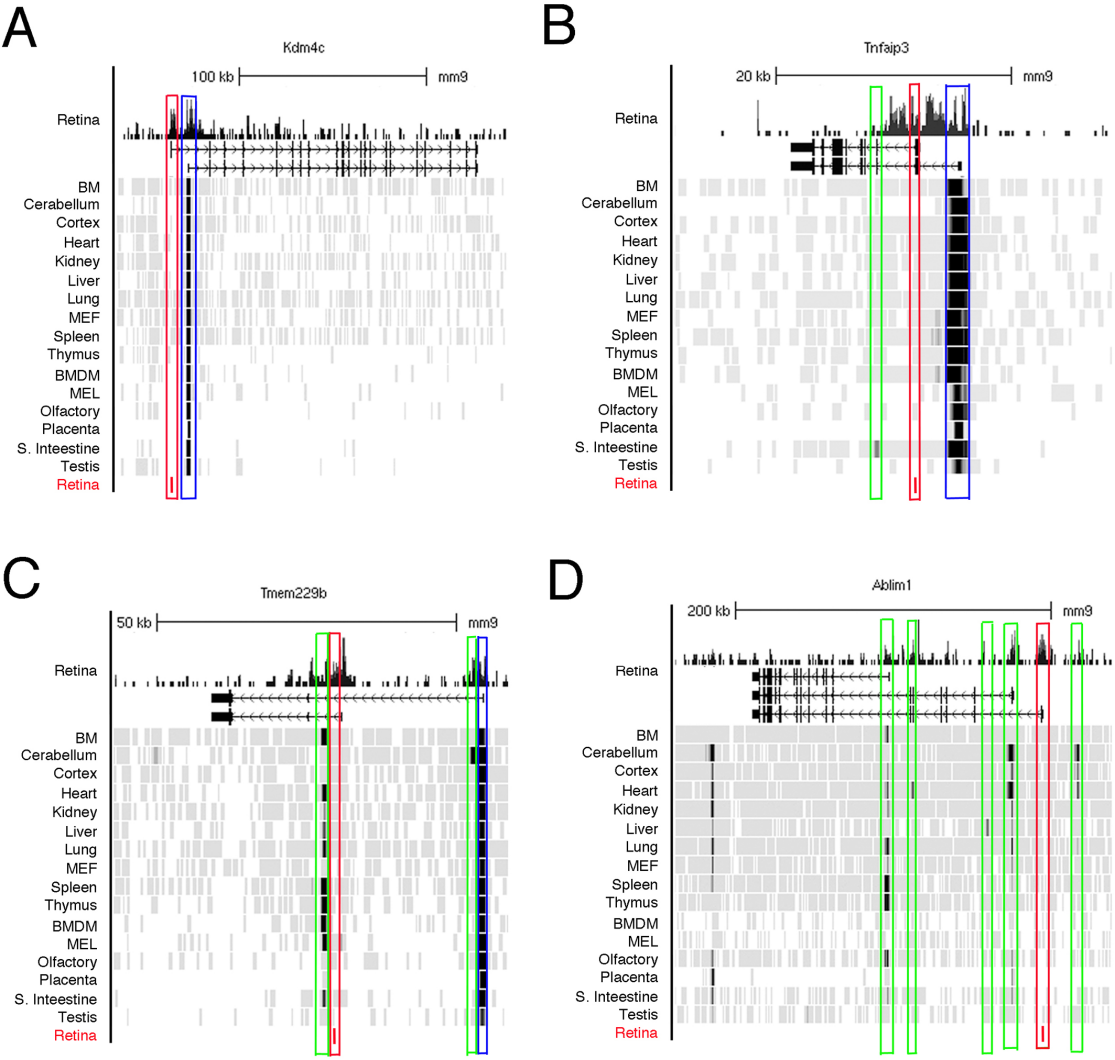
Use of alternative TSS for tissue specificity is universal. **A-D.** Combined genome-wide tracks of H3K4me3 accumulation (UCSC tracks for mouse browser mm9) for different mouse tissue and cells (bone marrow, cerebellum, cortex, heart, kidney, liver, lung, mouse embryonic fibroblasts, spleen, thymus, bone marrow derived macrophages, murine erythroleukemia cells, olfactory bulb, placenta, small intestine and testis) for the new rod genes: *Kdm4c* (**A**) (also see Fig 7C for this gene); *Tnfaip3* (**B**) (also see Fig 5H for this gene); *Tmem229b* (**C**) (also see Fig 6D for this gene); *Ablim1*(**D**) (also see Fig 3A for this gene). In each case common TSS is depicted as a blue box, rod TSS–as a red box and other tissue-specific TSS–as green boxes. For reference on the top of each figure panel presents the genome-wide track of H3K4me2 for retina at PN15 and gene position and locus structure.

Many of the genes that have a rod TSS and a common TSS are also expressed in other tissues. To explore TSS usage in variety of tissue we used accumulation of H3K4me3 as a marker of active TSS for several of these genes. We choose four new rod genes exhibiting both common and rod TSS. As shown in Fig 10, *Kdm4c*, *Tnfaip3*, *Tmem229b and Ablim1* show active transcription in many, but not all, tissues (Fig 10A–10D). The use of extra TSS varied considerably. For *Kdm4c* we detected a rod TSS but no additional TSS in any of the other tissue. *Tnfaip3* showed an additional TSS in small intestine that yields an even smaller transcript than the rod specific form. *Tmem229* showed 2 types of alternative TSS. One was restricted to cerebellum and other was used in multiple tissues. Ablim1 has multiple TSS. In addition to the rod TSS, two of the other TSS are used in multiple tissues (and were labeled common TSS in Fig 3). We also detected three additional potential TSS used by one or few tissues.

The mouse retina provides a good, though imperfect, model system to aid understanding of the human retina. As proof of concept, that rod TSS are conserved between human and mouse and that our approach to find tissue-preferential TSS is applicable to other organisms, we compared our data with RNA-seq data from human retina [57], which is available on UCSC Human Genome Browser. From our list of 107 genes—85% have same or very similar gene locus structure in mouse and human, and 81% of TSS in human demonstrate patterns of RNA-seq corresponding to the epigenetic profile in the mouse genome.

**Fig 11:**
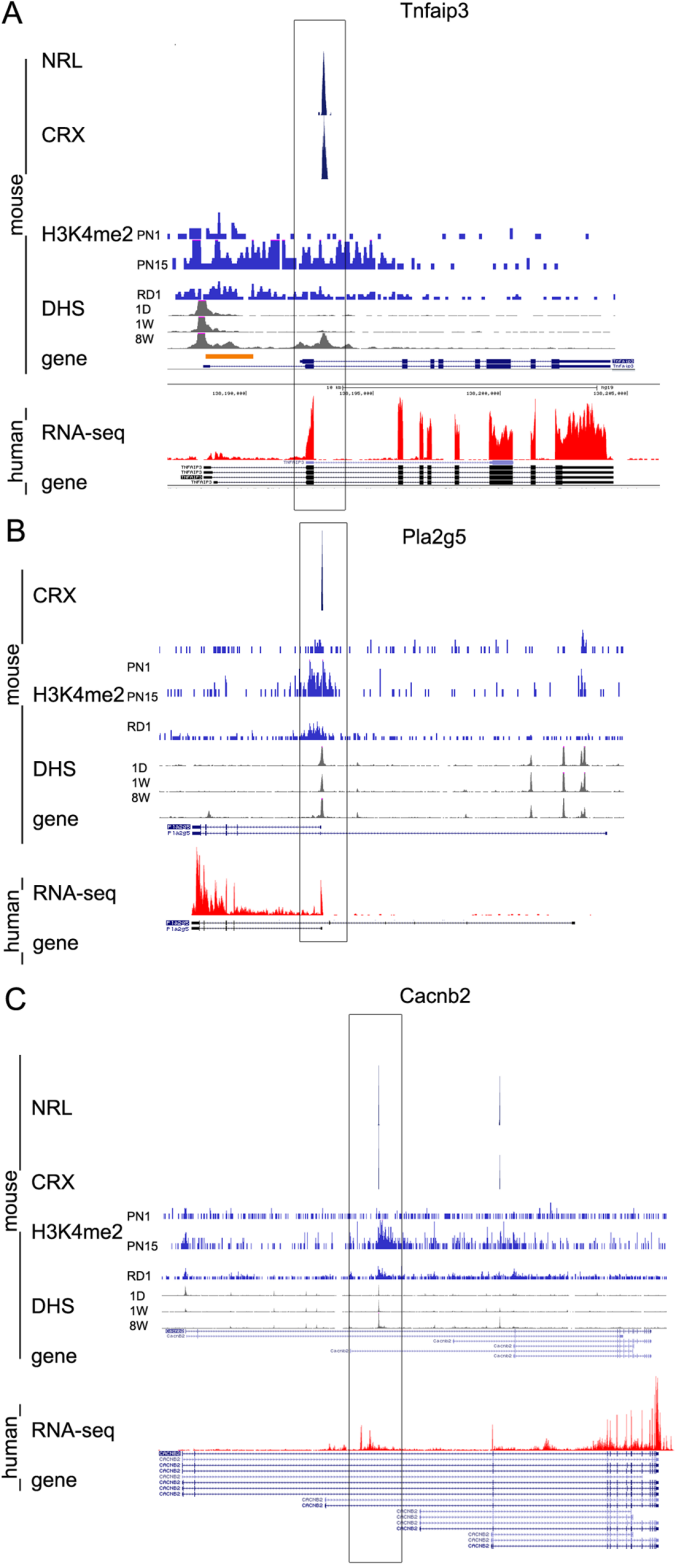
Rod TSS are conserved between human and mouse. **A, B, C.** Combined genome-wide tracks of chromatin features in mouse retina (developmental changes in H3K4me2 occupancy (PN1, PN15 and RD1 PN30), CRX-binding (PN56), NRL-binding (PN28), developmental changes in DHS (1D, 1W, 8W)) and RNA-seq for human retina for genes TNFAIP3 (**A**), PLA2G5 (**B**) and CACNB2 (**C**) genes. Predicted rod TSS that shows conservation between mouse and human depicted as a black box.

We found, that if the structure of the mouse gene locus is similar to that of the human, rod TSS could be easily distinguished from common TSS (Fig 11). For example, the TNFAIP3 gene is highly expressed in human retina from the same ATSS as in mouse (Fig 11A and Fig 5G), the same is true for PLA2G5, a gene from the RetNet list for mapped loci and genes causing retinal diseases that has been implicated in recessive benign fleck retina [58] (Fig 11B). RefSeq data shows that CACNB2 gene in mouse as in human has four alternative TSS. Epigenetic profiles in mouse retina pointed out an intron in the middle of the gene with increase in H3K4me2 accumulation and DHS in adult and CRX- and NRL-binding. We predicted, and confirmed, that *Cacnb2* has an additional rod TSS in the mouse (Fig 9A and 9B) that had previously been defined as an intron. The homologous region in human is also defined as an intron in RefSeq. The human retina RNA-seq data demonstrates an accumulation of RNA reads at this site, suggesting that in human too there is an exon produced from a novel rod TSS (Figs 11C and 9B).

All of these examples indicate that there is a rich landscape of alternative TSS yet to be explored and that the methods we have outlined can provide ways to identify them.

## Discussion

In addition to generating different transcripts, the use of alternative transcription start sites allows separate spatial and temporal regulation of transcription in different cell types and, when multiple TSSs are used in a single cell, allows separate control of constitutive and regulated levels of expression. In the present study, we have explored the properties and use of alternative TSS in the mammalian retina and specifically in one cell type, the rod photoreceptor. In the adult rodent retina, rods comprise at least 85% of the cells, making it easy to measure rod gene properties against a small background from other cell types. We have used the term rod TSS and rod gene in this work. We are confident that these transcripts are expressed in rods, based on the temporal expression patterns and significant loss of expression (p<0.0001), in the RD1 mutant retina [31].We cannot, however, exclude the possibility that some of these are also expressed in some other retinal cells, or in other tissues. The results shown in Fig 10 indicate that tissue restricted TSS may be used by a variable number of tissues or cell types, but not by all.

We previously showed that all previousky known rod genes (a total of 36) shared a specific developmental epigenetic signature, namely a de novo accumulation of H3K4me2 and a lack of H3K27me3 around the promoter, while some key precursors genes were losing H3K4me2 accumulation during development [31]. Inhibiting a key histone demethylase, LSD1, led to maintained levels of H3K4me2, sustained expression of precursor genes and a block in the expression of rod specific genes, confirming the active role of epigenetic modification in guiding developmental gene expression [59]. We used our rod epigenetic signature to screen the whole genome and found an additional 71 genes that were candidates for expression in rods.

We have now examined the TSSs of these genes and tested a group of chromatin and epigenetic features to explore and compare whether these characteristics can determine cell type specific TSSs, particularly in those genes with multiple TSSs. The features that we used were binding of the characteristic rod-specific transcription factors CRX [33] and NRL [32], developmental changes of DHS at the TSS [35], PolII binding [34] together with our developmental profiles of H3K4me2 accumulation.

CRX and NRL are transcription factors that have been well characterized as regulators of expression of rod specific genes involved in visual transduction. The 36 known rod genes, with the exception of *Cacna1f*, had CRX binding at one TSS. This association of CRX was also seen with the new rod genes, such that 76% of the genes with a single TSS and 73% of the rod TSS in genes with multiple TSS had CRX binding (Fig 5A). This is a strong correlation and emphasizes the importance of CRX in rod differentiation. It will be interesting to determine whether the other approximately 25% of genes use transcription factors are carrying out the same role as CRX.

NRL is also known to regulate the transcription of rod-specific genes but some known rod genes do not have measurable NRL binding. Overall, we found that 61% of the 72 rod genes with a single TSS showed NRL binding as did 56% of the rod TSS in genes with multiple TSS. This indicates that NRL is responsible for regulating the transcription of only a subset of rod genes and that other transcription factors, possibly other basic motif-leucine zipper family members, regulate the others.

DHS is a marker of open chromatin sites and, when present at TSS, is an excellent indicator of active transcription from that site. All known rod genes show a developmental increase in DHS that correlates with increased gene expression. A similar change in DHS was found for the new rod genes that had only one TSS. For those genes with multiple TSS it was clear that one behaved like a rod TSS and the other was active throughout development. This would suggest that DHS level can be a useful diagnostic of tissue-specific TSS when different ages can be compared.

In general, PolII binding can also help define rod TSS, but is less effective than the other markers. A greater proportion of genes in both the known rod and the new rod classes had no change in PolII binding between PN2 and PN25. The data set for PolII binding that available for retina and have been used here is based on ChIP-on-Chip assay and because of this have limited genomic coverage. Future studies based on ChIP-Seq methods and carried out at several developmental points could greatly improve our knowledge of PolII binding. Additionally, it is possible that PN2 is close to the time when many rod genes are beginning to assemble transcription complexes and so PolII is already bound, thus lowering the change. Determining PolII binding at an earlier time point, such as E17 might give better discrimination.

In many cases the TSS we have defined as “common” are active at high levels throughout development. One question that arises is whether they are used only by cells other than rods or whether they generate transcripts in rods as well as other cell types. Since the transcriptional level from common TSS in RD1 mice is comparable with the transcriptional level from common TSS in wild type mice (average ΔCt_rd1_ (Gapdh-gene) = 5.2; average ΔCt_wt_ (Gapdh-gene) = 5.1) it is likely that the common TSS are active in all retina cells and that the rod TSS serves to add additional transcripts to meet the high protein synthesis demand of these very active cells. Where the multiple TSS generate different proteins, this may provide a way of having multiple ways of regulating protein activity within a single cell type.

An alternative to our epigenetic approach to find new TSS is RNA-seq. Several RNA-seq data sets are available now for mouse retina [60–63], but in some instances comparison between these data sets show dissimilarities, possibly because of different genetic backgrounds or different methods for cell purification [62]. The power of our methodology is in comparing temporal epigenomic profiles at several developmental stages and combination of epigenetic signatures and TF binding data that can be used to define TSSs that are currently not recognized. In our study of 35 genes with multiple TSS, (6 are known rod-specific genes, whereas the other 29 identify new genes), we have tested and compared transcription for common and rod TSS at PN1 and PN15/adult retina for 11 of them. 9 showed the expected patterns (6—shown in Figs 3, 5, 6 and 7; 3 –data not shown), so it gives us an 18% false positive rate. The false negative rate is very small as from 98 control genes (Figs 2D; 5B; 6B) none had a rod pattern on their TSS. From our preliminary analysis of sites along mouse genome we suggest that many such TSS remain to be discovered. We tested the use of the new predicted TSS in three such examples (Fig 9). Each had a previously defined TSS (“common” by our criteria) and, using our analysis, each also has a rod TSS. In each case, we were able to verify a developmentally regulated transcript from this new TSS in retina that was lost in RD1 mice.

We found >80% concordance of mouse and human rod TSS, with biological confirmation of the human predictions from available RNA-seq data. This indicates that understanding the regulation of gene expression in the mouse will provide valuable information about the normal and diseased human retina, and also a good model to study therapeutic interventions that seek to modulate the expression of disease genes.

We have applied the approach of using a group of chromatin and epigenetic features to identify transcriptional complexity in one cell type, the rod photoreceptor. This approach gives the most confident results about transcription tissue specificity when chromatin markers at a particular TSS can be compared in different tissues (Fig 10) or at several time points in development, as we have shown here for H3K4me2 and DHS.

Based on our initial studies, we would predict that a similar approach can be used in other tissues and cell types to refine the current maps of TSS. While genes uniquely associated with the function of a cell, such as visual transduction pathway genes in rods, frequently have a single TSS, other genes with multiple TSS may represent the evolution of additional controls of the levels and nature of transcripts to better regulate cell-specific metabolism and function.

## Supporting information

S1 File Combined supporting figures. This file contains two figures labeled A, B.
(PDF)

S1 Table PCR primers. (XLSX)

S2 Table List of the 107 rod genes with single and multiple TSS. (XLSX)

S3 Table Epigenetic characteristic of rod genes TSS. (XLSX)

S4 Table List of genes for cluster 1 and 2. (XLSX)

S5 Table List of three groups of control genes. (XLSX)
