# Supplementary material for: Identification and prediction of alternative transcription start sites that generate rod photoreceptor-specific transcripts from ubiquitously expressed genes
Source: PLoS One. 2017 Jun 22;12(6):e0179230. doi: 10.1371/journal.pone.0179230 (PMC5480877; doi:10.1371/journal.pone.0179230)
Supplement: S1 File — This file contains two figures labeled A, B. (PDF) [file pone.0179230.s001.pdf]

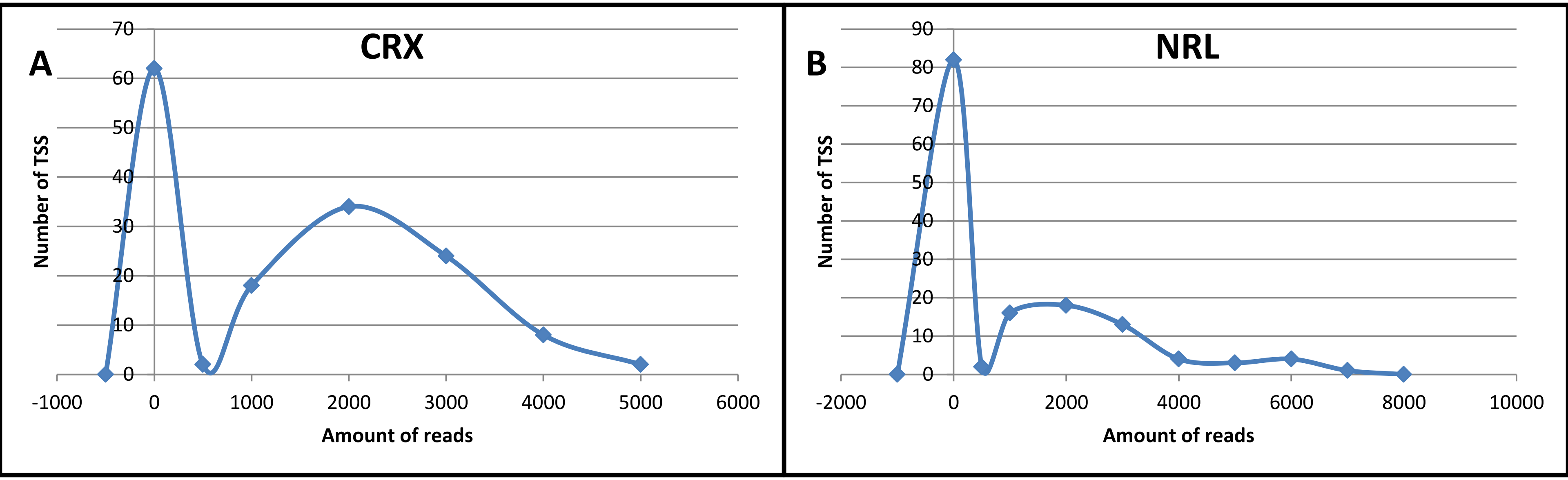

S1 file. Fig A and B. Bimodal binding of rod TF CRX (A) and NRL (B). A. For CRX -binding TSS were separated in 7 categories according to amount of reads in window TSS+/- 1000bp, counted in each category and plotted. Categories: 0; more than 0 but less 500 reads; more than 500 but less 1000; more than 1000 but less 2000; more than 2000 but less 3000; more than 3000 but less 4000; more than 4000 but less 5000. **B.** For NRL -binding TSS were separated in 10 categories according to amount of reads in window TSS+/- 1000bp, counted in each category and plotted. Categories: 0; more than 0 but less 500 reads; more than 500 but less 1000; more than 1000 but less 2000; more than 2000 but less 3000; more than 3000 but less 4000; more than 4000 but less 5000; more than 5000 but less 6000; more than 6000 but less 7000; more than 7000 but less 8000.
